# Impact of Fibrotic Tissue on Shear Wave Velocity in Thyroid: An* Ex Vivo* Study with Fresh Thyroid Specimens

**DOI:** 10.1155/2015/569367

**Published:** 2015-12-31

**Authors:** Takahiro Fukuhara, Eriko Matsuda, Yukari Endo, Ryohei Donishi, Shoichiro Izawa, Kazunori Fujiwara, Hiroya Kitano, Hiromi Takeuchi

**Affiliations:** ^1^Department of Otolaryngology-Head and Neck Surgery, Tottori University Faculty of Medicine, 36-1 Nishicho, Yonago 683-8504, Japan; ^2^Department of Pathology, Tottori University Faculty of Medicine, 36-1 Nishicho, Yonago 683-8504, Japan; ^3^Endocrinology and Metabolism, Department of Molecular Medicine and Therapeutics, Tottori University Faculty of Medicine, 36-1 Nishicho, Yonago 683-8504, Japan

## Abstract

We sought to elucidate the correlation between shear wave velocity (SWV) and fibrosis in thyroid by precisely assessing pathological structures inside 5 × 5 mm^2^ regions of interest (ROIs) of resected specimens, under conditions that excluded physical artifacts. The materials were unselected thyroid and lymph node specimens resected during thyroid surgery. Immediately after surgery, fresh unfixed thyroid and metastatic lymph node specimens were suspended in gel phantoms, and SWV was measured. Upon pathological examination of each specimen, the extent of fibrosis was graded as none, moderate, or severe. A total of 109 specimens were evaluated: 15 normal thyroid, 16 autoimmune thyroiditis, 40 malignant nodules, 19 benign thyroid nodules, and 19 metastatic lymph nodes. When all specimens were classified according to the degree of fibrosis determined by pathological imaging, the mean SWV was 1.49 ± 0.39 m/s for no fibrosis, 2.13 ± 0.66 m/s for moderate fibrosis, and 2.68 ± 0.82 m/s for severe fibrosis. The SWVs of samples with moderate and severe fibrosis were significantly higher than those of samples without fibrosis. The results of this study demonstrate that fibrosis plays an important role in determining stiffness, as measured by SWV in thyroid.

## 1. Introduction

Conventional elastography using manual compression evaluates stiffness of the target tissue relative to that of the surrounding tissue, whereas a recently developed elastography technique using acoustic radiation force impulse (ARFI) evaluates the local elastic characteristics of the target tissue itself [1]. In this method, a target region with fixed dimensions of 5 × 5 mm^2^ is identified as the region of interest (ROI), and shear wave velocity (SWV) is detected by a sonographic detection pulse in the ROI. The characteristics are expressed by the SWV of the tissue, which reflects tissue elasticity, as calculated using Young's modulus under the assumption that tissue density is 1 g/cm^3^ and has Poisson's ratio of 0.5 [2, 3]. Thus, shear wave elastography (SWE) is thought to be useful for quantitatively evaluating tissue hardness. However, it remains unclear what types of pathology affect tissue hardness.

In liver, many clinical studies of SWE have been conducted, and SWV is thought to be affected by liver fibrosis [4, 5]. Currently, shear wave elastography is frequently used to evaluate liver fibrosis.

Many clinical studies have evaluated the usefulness of SWE for differentiating benign versus malignant thyroid nodules [6–10], and a recent report described the usefulness of SWE for diagnosing autoimmune thyroiditis [11, 12]. In a previous clinical study, we investigated the correlation between SWV and the pathological structure of thyroid lesions and reported that the shear wave is accelerated as the extent of elastic fibrosis increases [1]. Other authors also pointed out the possibility of elevated SWV in fibrotic thyroid tissue [13, 14]. In past reports, however, the assessment of pathologies inside a 5 × 5 mm^2^ ROI, as reflected by measurement of SWV, may not be correct because the SWVs were measured* in vivo*. In particular, malignant tumors have multifaceted pathology, so it is possible that pathological structures change when the clinician's view is displaced by several millimeters. Indeed, SWVs measured in papillary thyroid cancer (PTC) tend to vary [1, 7, 9]. In light of these observations, in this study we sought to clarify the correlation between SWV and fibrotic pathology of thyroid specimens by precisely assessing the pathological structures inside a 5 × 5 mm^2^ ROI, as determined by SWV, under conditions that excluded physical artifacts. The results of this study indicate the most appropriate use of SWE in the thyroid.

## 2. Materials and Methods

### 2.1. Materials

Informed consent was obtained, and the study was performed in accordance with the ethical guidelines of the Helsinki Declaration. The ethics committee and the institutional review board of Tottori University approved the study protocol. The study period was from November 2011 to April 2014. The materials were unselected thyroid and lymph node specimens resected in thyroid surgery, at least 10 mm in diameter, so that they would encompass the entire ROI.

### 2.2. Methods

We used the Virtual Touch Quantification system (Siemens Medical Systems, Forchheim, Germany) to perform SWE using an ACUSON S2000 ultrasound system (Siemens Medical Systems) with a 9 MHz B-mode-ARFI combination linear transducer (ACUSON 2000; Siemens Medical Systems).

**Figure 1:**
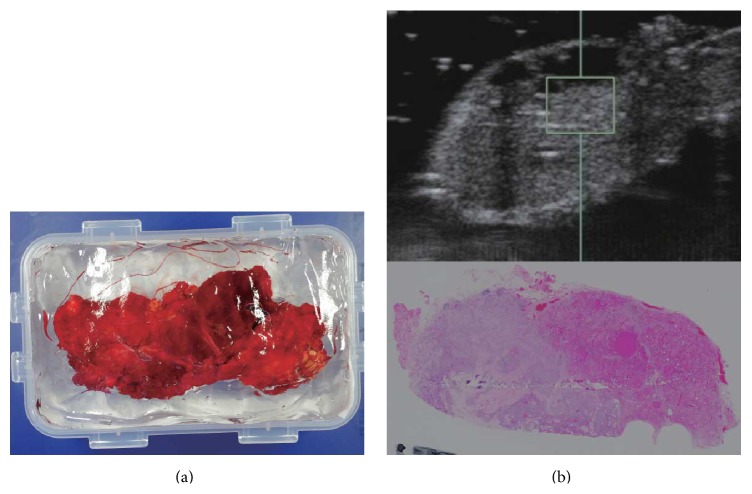
(a) Fresh unfixed specimens were suspended in gel phantoms, and the SWV was measured. (b) Histologic slides were created in the same plane used for ultrasonography imaging.

Immediately after surgery, fresh unfixed thyroid and metastatic lymph node specimens were suspended in gel phantoms and SWV generated by ARFI was measured (Figure 1). In each case, we scanned the specimens on B-mode, defined an ROI of 5 × 5 mm^2^, and measured SWV at the same point five times [15]. The 5 × 5 mm^2^ ROI was entirely within the thyroid specimen. Histologic slides corresponding to the ultrasonography imaging plane were created, and the degrees of fibrosis determined by imaging and SWV were compared.

### 2.3. Pathological Findings

**Figure 2:**
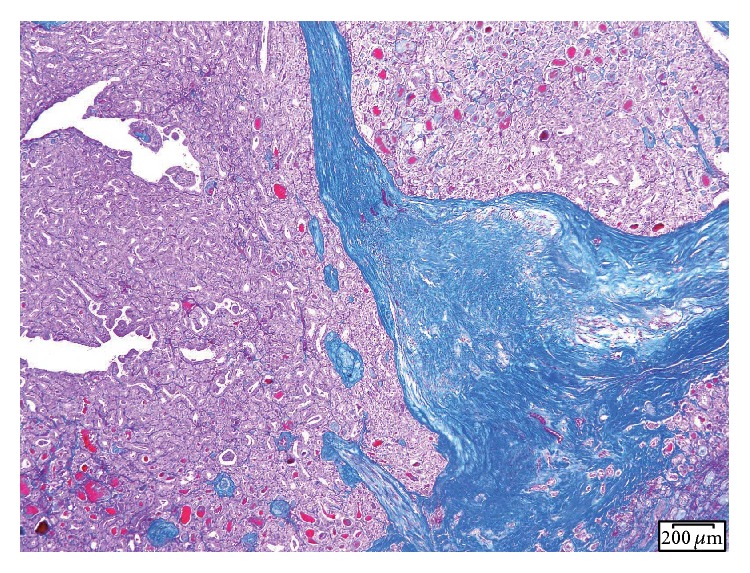
Collagen fibers were stained with Masson T stain.

All resected thyroid lesions and lymph nodes were analyzed pathologically. Collagen fiber on histologic slides was stained with Masson T stain (Figure 2), and the degree of fibrosis was assessed in 5 × 5 mm^2^ microscope fields in the plane used to measure SWV. Degree of fibrosis was classified into three groups: no fibrosis, moderate fibrosis, and severe fibrosis. The ImageJ software (version 1.48; Wayne Rasband, National Institutes of Health, Bethesda, MD, USA) was used to calculate the degree of fibrosis. Severe fibrosis was defined as extended fibrosis in over 50% of the area of 5 × 5 mm^2^ microscope fields, whereas moderate fibrosis indicated findings intermediate between no fibrosis and severe fibrosis. The correlation between SWV and the degree of fibrosis on pathological findings was evaluated.

### 2.4. Statistical Analysis

Statistical analysis was performed using SPSS software (version 22; IBM SPSS, Chicago, IL, USA). The SWVs of measured specimens (normal thyroid, autoimmune thyroiditis, benign nodule, malignant nodule, and metastatic lymph node) were expressed as the mean value ± standard deviation (SD) and compared using the Kruskal-Wallis test. The fibrosis grades of all targets were compared with their SWVs using the Kruskal-Wallis test. We then compared the SWVs between benign nodules and malignant nodules, depending on the fibrosis grade, using the Kruskal-Wallis test.

## 3. Results

**Figure 3:**
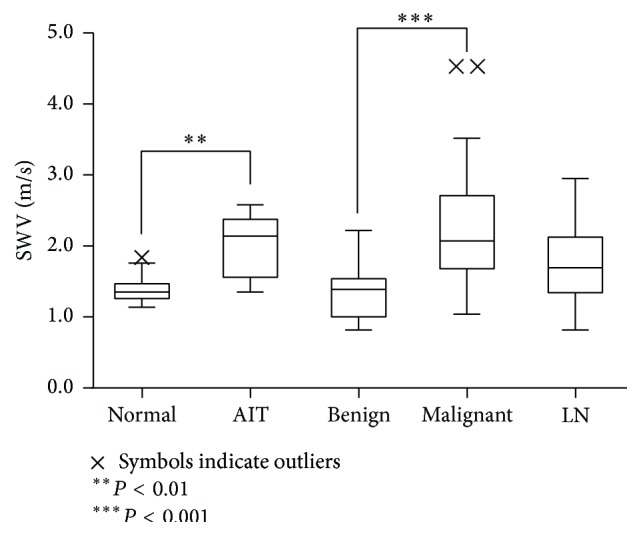
Mean SWV of each lesion.

A total of 109 specimens were evaluated: 15 normal thyroid, 16 autoimmune thyroiditis (AIT) (2 Hashimoto's thyroiditis and 14 Basedow disease), 40 malignant nodules (36 papillary thyroid carcinoma [PTC], 4 follicular carcinoma), 19 benign thyroid nodules, and 19 metastatic lymph nodes. Seven specimens were not measurable. The mean SWV was 1.40 ± 0.20 m/s for normal thyroid, 2.01 ± 0.42 m/s for autoimmune thyroiditis, 1.34 ± 0.37 m/s for benign nodule, 2.30 ± 0.82 m/s for malignant nodules, and 1.69 m/s for metastatic lymph node (Table 1, Figure 3). The SWVs of AIT and PTC were significantly higher than those of normal thyroid (*P* = 0.009 for AIT, *P* < 0.001 for PTC). Additionally, the SWVs of AIT and PTC were significantly higher than those of benign nodules (*P* = 0.002 for AIT, *P* < 0.001 for PTC). There was no difference in SWVs between normal thyroids and benign nodules.

**Figure 4:**
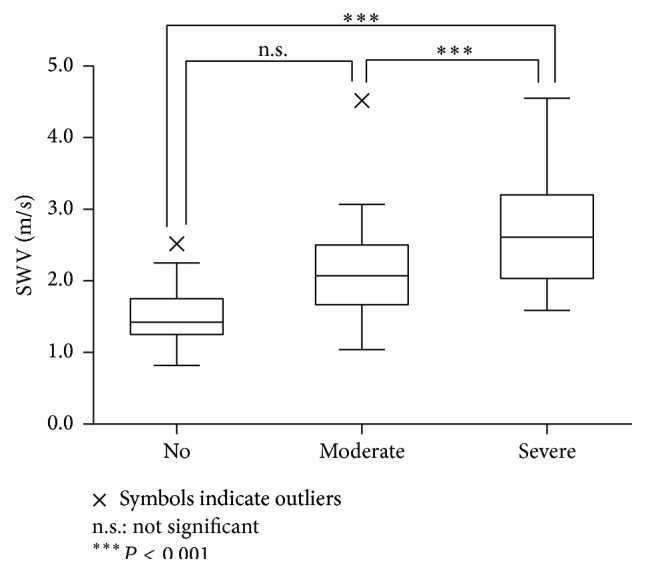
SWVs of specimens with no, moderate, and severe fibrosis when all specimens were classified according to the degree of fibrosis.

When all specimens were classified according to the degree of fibrosis determined by pathological imaging, the mean SWV was 1.49 ± 0.39 m/s for no fibrosis, 2.13 ± 0.66 m/s for moderate fibrosis, and 2.68 ± 0.82 m/s for severe fibrosis (Figure 4). The SWVs of samples with moderate and severe fibrosis were significantly higher than those with no fibrosis, but there was no difference in SWV between samples with moderate fibrosis and severe fibrosis. SWV increased with fibrosis severity (Spearman's *ρ* = 0.608).

**Figure 5:**
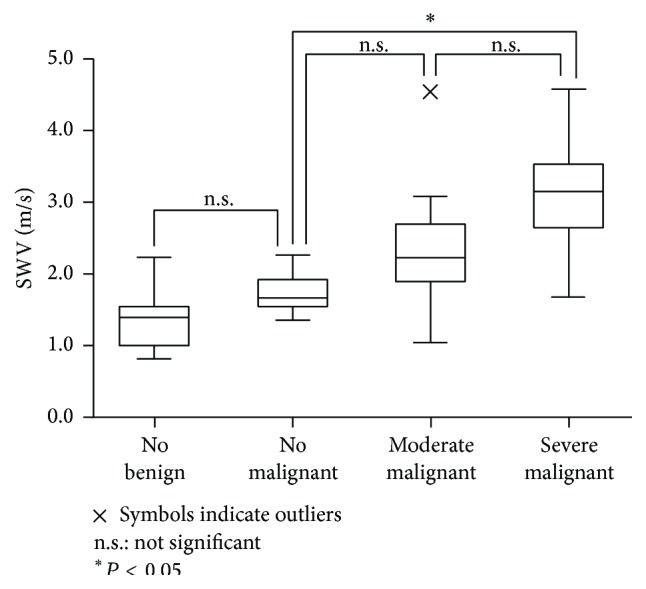
SWVs of thyroid nodules were compared according to fibrotic grade.

When SWVs of thyroid nodules were compared according to fibrotic grade, SWVs of malignant nodules with moderate or severe fibrosis were significantly higher than those of benign nodules with no fibrosis (*P* < 0.001) (Figure 5). There was no difference in SWVs between benign nodules without fibrosis and malignant nodules without fibrosis (Figure 5).

The pathologies of the 7 unmeasurable lesions were 3 calcifications, 2 cysts, and 2 heterogeneous tissues.

## 4. Discussion

We clarified the correlation between SWV and the precise pathological structure of targets inside the ROI by directly measuring resected thyroid specimens. The results revealed that fibrosis increased SWV, and that the variability of SWVs measured in malignant nodules was caused by variability in the fibrotic degree. Additionally, we showed that the results of SWV under conditions free of physical artifacts were similar to the results of previous clinical studies.

Shear waves are transverse waves that are very slow in comparison with acoustic waves and are therefore predicted to be easily affected by factors in the physiological environment such as carotid artery pulsation, respiratory movements, and reflections off the tracheal cartilage [1, 11, 16]. However, the degree to which such physical artifacts affect shear waves is unknown. The SWVs of each lesion, measured in resected specimens under conditions that excluded physical artifacts, were similar to the results of past clinical research in which SWVs were measured* in vivo* [1, 11, 12, 17–19]. The SWVs of AIT and PTC were significantly higher than those of normal thyroid or benign nodules, and there was no difference in SWVs between normal thyroids and benign nodules. Our results were also similar to the results of clinical studies [1, 9, 17–19]. The actual impact of the effect of the physiological environment upon SWV was judged to be negligible.

When the specimens were classified according to the degree of fibrosis determined by pathological imaging, and the SWVs of each fibrotic grade were compared, the results obviously showed that SWV increased with the severity of fibrosis. In this study, we observed no difference in SWV between moderate and severe fibrosis, probably because the moderate grade included a very wide range, from slight to 50%. There was no difference in SWV between normal thyroid and benign nodules, although the cell density obviously differed between these types of samples. As in a past study, the effect of cell density on SWV was thought to be small [1]. SWVs at solid cell patterns did not differ between normal controls, benign nodules, and PTCs. However, SWVs in fibrotic regions were significantly higher than those in nonfibrotic regions, and the SWV increased with fibrosis severity.

Several studies of SWV in the thyroid region have reported the usefulness of this technique for differentiating between benign and malignant thyroid nodules [8, 9, 20]. However, the SWVs of malignant nodules were variable. When we classified the malignant nodules according to the degree of fibrosis, the results revealed that the SWVs of malignant nodules differed significantly according to the degree of fibrosis. The SWVs of malignant nodules without fibrosis were not different from those of benign nodules. In general, benign nodules often have some fibrosis at the capsule. However, no fibrosis was observed in 5 × 5 mm^2^ microscope fields of benign nodules, because in this study we measured SWV inside the nodules and placed the 5 × 5 mm^2^ ROI entirely within the thyroid specimens. Fibrotic changes occur in most of PTC. Therefore, SWE may be helpful as an auxiliary diagnostic method for differentiating between benign and malignant nodules by measuring within an ROI encompassed within the thyroid nodule.

In this study, the SWVs of resected specimens were not measurable when the pathologies were calcifications, cysts, or heterogeneous tissues. Some authors also reported that these lesions were unmeasurable [1, 17, 18]. In the cases of calcifications or cysts, we believe that ARFI was not transmitted (due to reflection or absorption, resp.), and consequently no shear wave was generated. On the other hand, heterogeneous tissues were probably unmeasurable due to errors caused by the measurement principle of the ACUSON S2000 ultrasound system [1].

The findings of this study have potential clinical impact. In particular, measurement of SWV may be useful for choosing biopsy sites or predicting the extent of cancer invasion.

There were some limitations in this study. First, the efficiency of propagation of ARFI may differ in gel and* in vivo*. Consequently, the degree of generated SW may also differ between these conditions. Therefore, the proportion of unmeasurable samples was lower than in an* in vivo* study [1]. Second, the SWVs were measured in resected thyroid specimens under conditions very different from those that arise* in vivo*; in particular, there was no blood flow, and it is possible that this influenced the SWVs. Third, it is possible that unknown factors other than fibrosis and cell density influence SWV.

## 5. Conclusions

In this* ex vivo* study, we created histologic slides corresponding to the ultrasonography imaging plane. Comparison of the imaging and SWV findings revealed that the SWV of the target tissue was affected by its pathology. Fibrosis played an important role in the stiffness, measured by SWV, in thyroid tissue.

## Figures and Tables

**Table 1:** Number and characteristics of specimens of each lesion.

		Normal	AIT	Benign nodule	Malignant nodule	Lymph node
*N*		15	16	19	40	19

Measurable		15	16	19	36	16

SWV (mean ± SD)		1.40 ± 0.20	2.01 ± 0.42	1.34 ± 0.37	2.30 ± 0.82	1.72 ± 0.57

Fibrosis	No	15	3	19	12	9
	Moderate	0	8	0	20	8
	Severe	0	5	0	8	2

Note: SWV, shear wave velocity; SD, standard deviation; AIT, autoimmune thyroiditis.
